# Synthesis, Characterization and Adsorptive Performances of a Composite Material Based on Carbon and Iron Oxide Particles

**DOI:** 10.3390/ijms20071609

**Published:** 2019-03-31

**Authors:** Vasile Mînzatu, Corneliu-Mircea Davidescu, Petru Negrea, Mihaela Ciopec, Cornelia Muntean, Iosif Hulka, Cristina Paul, Adina Negrea, Narcis Duțeanu

**Affiliations:** 1 Politehnica University Timisoara, Faculty of Industrial Chemistry and Environmental, Victoria Square, no. 2, Timisoara 300006, Romania; vasile.minzatu@student.upt.ro (V.M.); mihaela.ciopec@upt.ro (M.C.); cornelia.muntean@upt.ro (C.M.); cristina.paul@upt.ro (C.P.); adina.negrea@upt.ro (A.N.); narcis.duteanu@upt.ro (N.D.); 2 Engineering, Research Institute for Renewable Energy, Politehnica University of Timisoara, Timisoara 300006, Romania; iosif.hulka@upt.ro

**Keywords:** composite, iron oxide, soluble starch, adsorption, arsenic removal

## Abstract

The aim of this paper was to produce a new composite material based on carbon and iron oxides, starting from soluble starch and ferric chloride. The composite material was synthesized by simple thermal decomposition of a reaction mass obtained from starch and iron chloride, in an inert atmosphere. Starch used as a carbon source also efficiently stabilizes the iron oxides particles obtained during the thermal decomposition. The reaction mass used for the thermal decomposition was obtained by simultaneously mixing the carbon and iron oxide precursors, without addition of any precipitation agent. The proper composite material can be obtained by rigorously adhering to the stirring time, temperature, and water quantity used during the preparation of the reaction mass, as well as the thermal regime and the controlled atmosphere used during the thermal decomposition. Synthesized materials were characterized using thermogravimetric analysis, X-Ray Diffraction (XRD), scanning electron microscopy (SEM), and Fourier transform infra-red spectroscopy (FT-IR). The performances of the obtained material were highlighted by studying their adsorbent properties and by determining the maximum adsorption capacity for arsenic removal from aqueous solutions.

## 1. Introduction

Iron oxides are widely used in chemical synthesis, in the wastewater remediation process, as well as in the treatment of drinking water. Beside these examples, iron oxides are used as catalysts in various industrial processes [1,2], as adsorbent for removal of some pollutants [3,4,5], or as extractants used to improve the adsorbent properties of some synthesized or chemically functionalized adsorbent materials [6,7,8,9]. Catalysis and adsorption processes are based on mass transfer phenomena taking place at the solid–liquid or solid–gas interface; to increase the efficiency of these phenomena it is necessary for the iron oxides to have a large specific surface area. The specific surface area of adsorbent materials can be increased by reducing as much as possible the size of the particles. In order to obtain a proper specific surface area, the size of the produced iron oxide particles must be in the range of hundreds of nanometers [10,11].

However, the actual usage of such small particles is really difficult because it is a hard job to separate such small particles from the reaction environment. Therefore, an alternative of this would be to attach such nanoparticles to different macro-sized supports, which must be chemically, mechanically and thermally stable. One of the most utilized supports in real application is represented by carbon, in the form of graphite or activated carbon, which presents a large surface area, as well as being a relatively cheap and environmentally friendly material. A green way to produce such a support material can be represented by the pyrolysis of various organic compounds with large molecular size, such as cellulose, starch, and bio-polymers [12]. 

The activity of such material can be increased by attaching iron oxide particles onto the support material, a complex process which can be accomplished in several stages. Such a process has a relatively high resources consumption, and at the same time it is difficult to implement physical–chemical methods [13,14,15].

**Figure 1:**
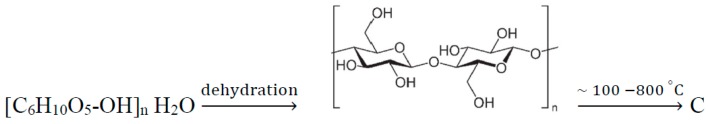
Mechanism of thermal decomposition of starch.

Surprisingly until the 2000s no natural polymer had been considered for the production of carbon based support materials [16]. Since then materials such as alginate, chitosan, cellulose, or other polysaccharides have been used as raw materials for carbon structure production. Starting from that time, it has been possible to produce carbon particles with different hydrophilicity by using a simple and economical process of thermal decomposition of starch between 100 °C and 800 °C [17,18]. By controlling the decomposition temperature it is possible to control the number of oxygen containing groups formed on the surface of the produced material. The starch thermal decomposition process is schematically presented in Figure 1.

Simultaneously, the thermal decomposition temperature controls the particle size and stability, as well as the pore size and volume. One other factor which controls the pores form and volume is represented by the natural ability of amylose and amylopectin chains from natural polymers (e.g.starch) to assemble in a supramolecular lamellar structure with nanometric dimensions, presenting crystalline and amorphous areas [17]. At the molecular level the thermal decomposition of starch takes place in several stages. In the first stage, at 200 °C dehydration occurs, catalyzed by intermolecular acidic groups leading to etheric groups, fixing in this way the structure of the porous material. At higher temperature (300 °C), the residual hydroxylic groups condense and sp2 hybridization produces molecule linearization. This transformation leads to an increase of the specific surface and microporosity, forming different 3D structures such as cylinder sections. Simultaneously it is possible that the decomposition of the melted phase takes place. Starting from 550 °C an extended system of double conjugated bonds, leads to a spatial rearrangement of the structure, similar to the 2D graphitic structure, thereby increasing the number of lamellar pores and the system microporosity. Further increase of temperature completes the transformation leading in this way to a mesoporous carbon structure [19,20].

**Figure 2:**
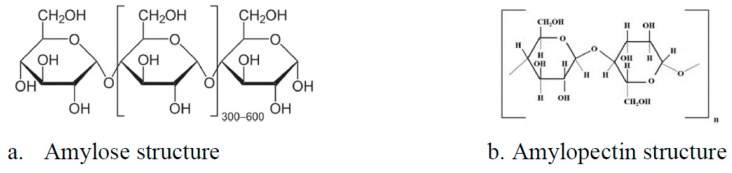
Starch structure.

Zhang et al. [21] and Liang et al. [22] demonstrate that starch can stabilize magnetite particles at the nanoscale level, and the produced composite material becomes an effective adsorbent material for in situ remediation of arsenic contaminated soils. Soluble starch (an environmentally friendly compound) is a complex polysaccharide composed of two different polymers amylose (Figure 2a) and amylopectin (Figure 2b), found in variable proportions in the starch structure [23]. 

Amylose is a natural polymer with a molecular mass between 10,000 and 340,000 Daltons and is formed from maltose units linked by 1-4 α glyosidic bonds, while having a linear structure, with a spiral form. Each spiral consists of six fragments of glucopyranosyl groups. Amylose is a compound relatively soluble in cold water, which does not form a sticky dough with hot water. 

The structural units of amylopectin are maltose and isomaltose having a branched structure, an amylopectin chain is formed from α-d-glucopyranose linked by 1-4 and 1-6 α-glycosydic bonds. This compound is not soluble in cold water, but can be dissolved in hot water which is responsible for dough formation [24].

In acidic environment hydrolysis of soluble starch occurs by breakage of the glycosidic bonds forming glucose molecules. Experimental data confirm that the acidic hydrolysis of starch takes place preferentially in amorphous areas, areas rich in amylose, while areas rich in amylopectin are less susceptible to hydrolysis [25].

Starch can be modified by using acidic media with slow boiling, forming oxidized starch, crosslinked starch, partially esterified or etherified starch, or even starch converted to cationic derivatives. Production of new bonds occurs at the surface of the starch dough, which reacts with bi- or poly-functional specific reagents like phosphorus oxychloride (POCl_3_), sodium trimethyl phosphate, epichlorohydrin, acetic acid, adipic acid [26]. 

Furthermore, acidic hydrolysis is influenced by several factors such as: time, temperature, and starch concentration. Thus, with a short hydrolysis time it was observed that the amylose hydrolysis take place predominantly without the rupture of bonds from the starch structure, whereas with a long hydrolysis time the rupture of starch bonds occurs leading to higher crystallinity of the compound [25]. Another parameter with higher influence is temperature; when the hydrolysis takes place at low temperature (room temperature), the degree of hydrolysis has extremely low values. Also the hydrolysis temperature cannot be really high, because at the temperature interval of 57–60 °C the starch gelatinization process occurs [25]. So, as a result of acidic starch hydrolysis the reaction mass contains glucose as the hydrolysis product, along with partially hydrolyzed and unhydrolyzed starch particles. All chemical and biochemical starch transformations occur in one stage, the transformation degree being dependent on the number of transformed covalent bonds (broken or new formed bonds). Therewith, higher importance is played by the physical transformations which are not responsible for covalent bond modification, being responsible for the increase of the starch molecule complexity. As a consequence, the starch transformation based on dehydration leads to carbon particles and has become the most common method for production of carbon nanoparticles [17]. 

As a consequence of higher affinity or iron ions for arsenic it is important to design, develop, and produce environmentally friendly adsorbents loaded with iron oxide particles for water remediation. Iron oxide particles can be produced through different methods such as the following: the sol-gel method [27], by precipitation [28], the combustion method [29], the Pechini method [30] etc. 

Traditionally the production of composite materials based on carbon and iron oxides was a two stage synthesis: in the first stage the activated carbon and iron oxide particles were produced, followed in the second stage by the thermal treatment of a physical mixture of them [31]. Iron oxide particles synthesis is possible by using different precipitation agents such as: sodium hydroxide [21] or ammonia [32], the particle size being efficiently stabilized by the starch mass [21,22,32].

The aim of the present study was to produce a new composite material based on carbon and iron oxide, with application for arsenic removal. As precursors used for the preparation of the desired composite material, soluble starch and pure iron chloride were used. Synthesis of the composite material was also a two stage process; stage one, consisting of the production of the reaction mass, using by a mixture of raw materials used as precursors (starch and iron chloride), followed by the thermal decomposition in a nitrogen atmosphere (second stage). Employing such a synthesis route, the production of iron oxide particles is then possible without adding any precipitation agents. After production, the new composite material was characterized and tested as an adsorbent for arsenic removal from aqueous solutions.

## 2. Results and Discussions

### 2.1. Synthesis of the Composite Material Based on Carbon and Fe_3_O_4_ Particles 

Composite material was synthesized as described in Section 3. The obtained experimental data confirm that it is possible to obtain smaller iron oxide particles (nano-sized) when the reaction mass is stirred for a relatively long time. Also, it was confirmed that the starch could be kept in the reaction mass in the form of paste only if the temperature was maintained at less than 60 °C.

Production of the desired composite material using starch and iron chloride as precursors can be understood if we consider that by dissolution of anhydrous iron chloride in water a hydrolysis process takes place.

By simple mixing of starch with water a starch dough is produced without any changes in the starch structure. By dissolving anhydrous iron chloride in water, iron (III) cations and chloride anions are produced, with a concomitant decrease of the solution pH.

FeCl_3_ → Fe^3+^_(aq)_ + 3Cl^−^_(aq)_ (1)

Hydrolysis behavior of iron (III) cations in diluted solutions at low pH values leads to the formation of iron oxy-hydroxides and iron hydroxides according to the mechanism below [27,33]:

Fe[(H_2_O)_6_]^3+^ + H_2_O → Fe[(H_2_O)_5_OH]^2+^ + H_3_O^+^ (2)

Fe[(H_2_O)_5_OH]^2+^ + H_2_O → Fe[(H_2_O)_4_(OH)_2_]^+^ + H_3_O^+^ (3)

Fe[(H_2_O)_4_(OH)_2_]^+^ + H_2_O → Fe[(H_2_O)_3_(OH)_3_] + H_3_O^+^ (4)

Fe[(H_2_O)_6_]^3+^ + _3_H_2_O → Fe[(H_2_O)_3_(OH)_3_] + _3_H_3_O^+^ (5)

As a consequence of hydrochloric acid production, acidic starch hydrolysis occurs according to the following reaction:

(6) $$–(C_{6}H_{10}O_{5}){–}_{n}+{}_{n}H_{2}O\overset{\mathrm{acid}\;\mathrm{hydrolysis}}{\to }{}_{n}C_{6}H_{12}O_{6}$$

Glucose molecules produced during starch acidic hydrolysis replace water molecules from the structure of iron aqua complexes, forming new complexes with glucose. Formation of such complexes prevents further condensation reaction between the aqua complex particles stabilizing it and therefore limiting their size (Equation (7)) [21,22].

Fe[(H_2_O)_3_OH)_3_] + C_6_H_12_O_6_ → Fe[C_6_H_12_O_6_ (OH)_3_] + _3_H_2_O (7)

Iron hydroxy-aqua complexes formed during the hydrolysis of iron chloride can participate in the next step in a condensation reaction, giving as a result Fe–O–Fe bond formation during thermal treatment of the reaction mass [27].

Based on experimental data obtained during preliminary attempts [34], the optimum conditions were established for synthesis of the desired composite material at different temperatures. So, when the synthesis was conducted at 45 °C, the reaction mass had to be stirred for 240 min (S2), at 55 °C the reaction mass had to be stirred for 45 min (S1), and at 58 °C the reaction mass had to be be stirred for 20 min (S3). The produced paste was dried for 24 h at 50 °C, ground, and then subject to a thermal decomposition process in a controlled atmosphere (nitrogen atmosphere) at 600 °C for 6 h.

The conditions used for the synthesis of the produced composite materials are presented in Table 1.

After the synthesis the material was characterized by physical–chemical methods using FTIR, XRD, and SEM in order to establish the optimum condition for the compound production and in order to characterize the structure of the obtained adsorbent material.

### 2.2. Physical-Chemical Characterization of the Synthesized Composite Material

#### 2.2.1. Thermal Gravimetric Analysis (TGA)

**Figure 3:**
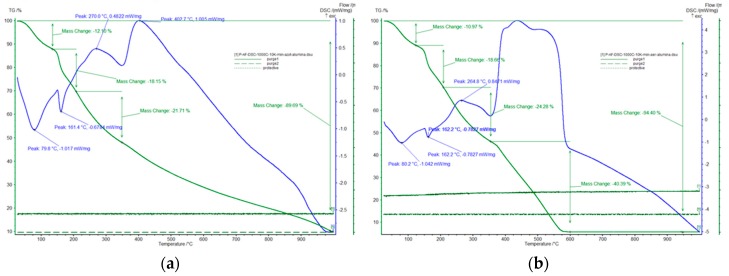
Thermal-gravimetric analysis of the synthesized material (AM-Fe) in a nitrogen atmosphere (**a**) and in air (**b**).

TGA curves depicted in Figure 3 were recorded at a heating speed of 5 °C per minute in a nitrogen and air atmosphere.

Analyzing the TGA curves depicted in Figure 3a (curve recorded in a nitrogen atmosphere) the following processes were identified: when the temperature is increased to 120–130 °C a mass loss associated with sample dehydration can be observed. Further increase of temperature until 200–220 °C is associated with removal of water molecules contained in the formed aqua cations. A further temperature increase leads to starch decomposition, a process which takes place between 250–360 °C. Such a decomposition process is the result of the rupture of glycosidic bonds, which leads simultaneously to a carbon structure formation with release of several volatile compounds—CO, CO_2_, aldehydes, furans [23]. Between 280–500 °C the formation of the iron oxides (magnetite, maghemite) takes place from used iron precursors.

When the thermal decomposition curves were recorded in air (curves depicted in Figure 3b) we can observe that alongside the changes observed between 100 and 400 °C (already presented) one new highly exothermic process occurs when the temperature is located between 500 and 600 °C, a process associated with carbon burning, resulting only in iron oxide as residue.

Based on data obtained from the analyzed thermogravimetric curves it was established that the thermal treatment of “starch–iron chloride” paste must be carried out in a controlled nitrogen atmosphere using a temperature of 400 °C. These conditions were chosen in order to preserve the carbon structure of the obtained material. Experimental data confirm that iron oxide was obtained when the sample was heated by using a relatively low speed ramp (5 °C min^−1^), this speed ramp also avoided destruction of the carbon structure.

#### 2.2.2. Fourier Transform Infra-Red (FT-IR) Spectroscopy Analysis

**Figure 4:**
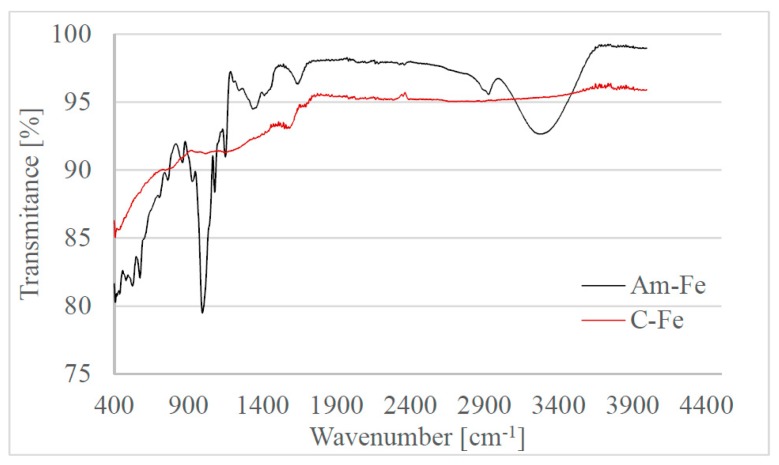
FTIR spectra for the synthesized material before thermal decomposition (Am-Fe) and after thermal decomposition (C-Fe).

FT-IR spectra were recorded for the initial material and for the material obtained after thermal decomposition. The recorded spectra are depicted in Figure 4.

From the data depicted in Figure 4 we can observe: that in the FT-IR spectra of C-Fe material (material obtained after the thermal decomposition) the peaks associated with the presence of starch are not present (peaks located between 3500–3000 cm^−1^ and 1200–800 cm^−1^); the presence of some peaks specific for Fe–O bonds, peaks are located in the interval 1000–450 cm^−1^; an increase of the C–C, C–O and Fe–O specific bands is located between 2000 and 1000 cm^−1^; and that by comparing the FT-IR spectra of Am-Fe material with the spectra recorded for pure starch, we notice that the starch IR vibrations are shifted towards higher wave numbers having lower intensities [35].

Recorded FT-IR spectra confirm that thermal decomposition of Am-Fe material leads to the formation of a carbon structure which contains iron oxide particles bonded onto the carbon surface by oxygen bonds.

#### 2.2.3. X-Ray Diffraction Analysis (DRX)

**Figure 5:**
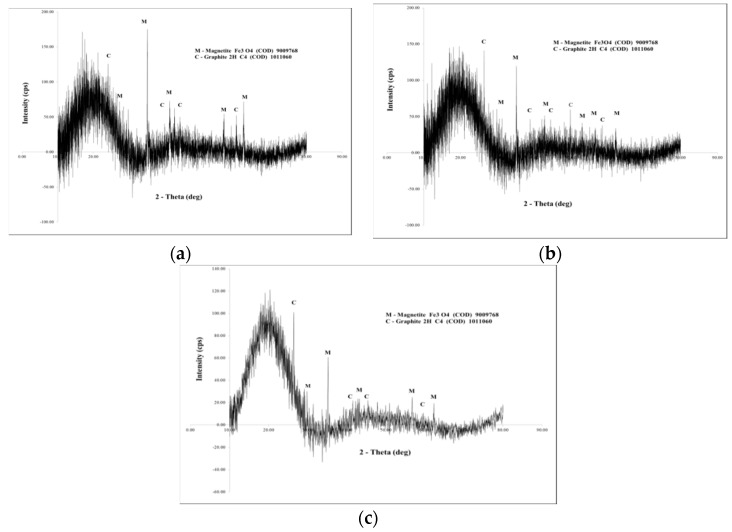
X-ray diffraction spectra for samples: (**a**) S1; (**b**) S2; (**c**) S3.

The recorded XRD spectra obtained for three synthesized materials are depicted in Figure 5. By analyzing the presented spectra we can observe that the synthesized materials are heterogeneous containing two different phases: carbon and magnetite [36,37].

From spectra depicted in Figure 5 we can observe a similar behavior for all analyzed samples, which leads us to conclude that the starch decomposition during thermal treatment occurs with formation of a carbon crystalline network concomitant with formation of Fe_3_O_4_ particles. By looking at these spectra, we can also conclude that the stirring time has no effect over the species that appear in the final product.

#### 2.2.4. Scanning Electron Microscopy Analysis SEM

**Figure 6:**
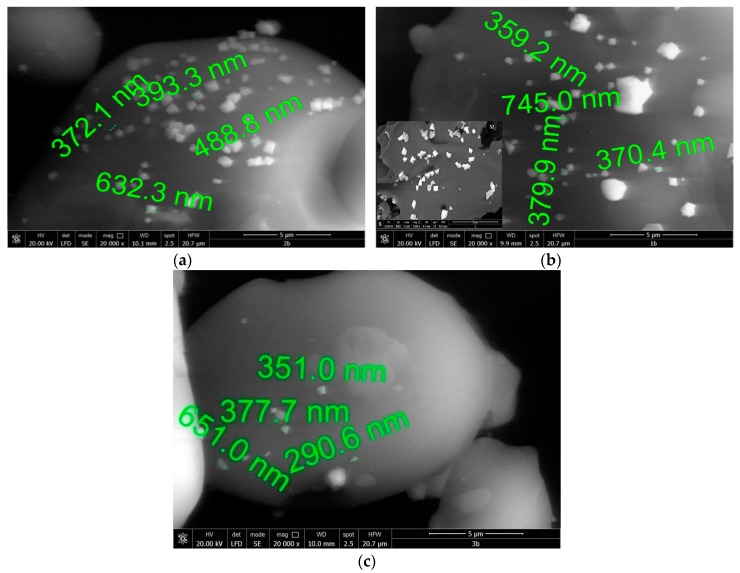
Scanning electron microscopy imagines for the samples: (**a**) S1; (**b**) S2; (**c**) S3.

Recorded SEM micrographs are depicted in Figure 6 and were analyzed in order to obtain information about the material morphology and the magnetite crystal distribution on the carbon particles.

Based on data presented in the SEM pictures we can observe that the magnetite particles have a relative uniform size. From Figure 6a (sample 1) it can be observed that the magnetite particles present dimensions around 450 ± 150 nm, in comparison with the other two samples obtained with lower stirring time (sample 2, Figure 6b). In this second case we can observe that the magnetite particles size is close to the size of particles from S1, but with uneven distribution on the surface of the carbon particles. Based on that it can be concluded that the increase in stirring time leads to lower size and uniform distribution of the magnetite particles on the carbon particles surface. This behavior can be explained if we take into account that the increase of stirring time leads to a higher hydrolysis degree of the starch concomitant with incorporation of hydroxy-aqua ions into the starch structure, leading to a more homogenous material after thermal treatment.

Data obtained for sample 3, which was obtained using a low stirring time (20 min) but with the highest decomposition temperature (58 °C), confirm that a heterogeneous material containing few magnetite particles with higher size distribution was produced. Increasing the stirring time at this temperature has no beneficial result due to the occurrence of gelatinization which favors the separation of iron oxides from the starch particles, leading to partial separation during the paste drying.

#### 2.2.5. Sorption Performances of the Synthesized Material

An important application of the newly synthesized materials is represented by their usage as adsorbents for removal of different pollutants the high affinity of arsenic ions for iron being well known [3,4,5,38,39,40,41,42,43,44,45]. In the present paper the performance of new synthesized materials for arsenic removal from water solutions was studied, by determining the maximum adsorption capacity of each produced adsorbent material. The adsorption experiments were carried out using the methodology presented in Section 3.2.

Taking into account the procedure, from each sample a quantity of 0.1 g was measured, which was mixed with a set of 25 mL solutions containing variable arsenic concentrations. The adsorbent material was kept in contact with the arsenic solution for one hour in a thermostatic shaker bath at 200 rotations per minute. After one hour, the solution was filtered and the residual arsenic concentration was measured in the solution obtained after the adsorption process was performed.

The maximum adsorption capacity obtained from the experimental studies is presented in Table 2 in comparison with the data obtained from scientific literature.

On analyzing data presented in Table 2 we can observe that the maximum adsorption capacities obtained when the newly produced composite materials were used are similar or superior to the maximum adsorptions capacities presented in the scientific literature. Also, from the obtained maximum adsorption capacities it can observed that there is a correlation between the maximum adsorption capacity and the magnetite content in the adsorbent material. So, the highest maximum adsorption capacities were obtained for samples S1 and S2, samples which had the highest magnetite content. The difference observed between the maximum adsorption capacities obtained for samples S1 and S2 can be explained if we consider the difference between the magnetite particle size and the sample homogeneities.

When a magnetite content decrease was observed then the maximum adsorption capacity of the newly produced adsorbent material decreased. In the case of sample S3, the maximum adsorption capacity was 26 μg As(V) per gram of adsorbent, which is seven times lower than the maximum adsorption capacity obtained for sample S2. Also, based on data presented in Table 2 we can observe that the newly produced composite material designated as sample S2 presents an adsorption capacity higher than the majority of the adsorbents described in the literature, even higher than some natural materials.

## 3. Materials and Methods

### 3.1. Preparation and Characterization of Material

The composite material was synthesized using the following route: 20 g of soluble starch (Merck—designated as Am) was mixed with 25 mL of distilled water, in triplicate). Each obtained sample was heated at a different temperature, 45, 55, and 58 °C. When the starch mass attained the desired temperature, over it under continuous stirring a solution of ferric chloride (Merck) was poured with a concentration of 33.33 % (2.5 g of anhydrous ferric chloride was dissolved in 5 mL of distilled water). The reaction mass was stirred continuously for various time intervals (20, 45, and 240 min) until transformed into a paste. These quantities of precursors were calculated in order to obtain a final ratio of Fe:C equal to 1:10. This ratio was chosen because the experimental data showed that the usage of higher quantities of iron chloride was not recommended because it leads to starch gelatinization, simultaneously it was proved that smaller ratio leads to a composite material with a lower efficiency in various applications [34].

Raw material used for thermal decomposition and the composite material obtained (designed as Am-Fe) were characterized through thermogravimetric analysis (using the thermal analysis scale Netzch STA 449C, using a heating speed of 5 °C min^−1^, in nitrogen atmosphere), X Ray Diffraction (using a Rigaku Ultima IV XRD diffractometer), scanning electron microscopy (SEM—using a Quanta FEG 250 scanning electron microscope), and Fourier transform infrared spectroscopy (FT-IR—using a Bruker Platinum ATR-QL-Diamond spectrometer).

### 3.2. Sorption Performances of the Obtained Material

Synthesized composite material was used as adsorbent material for arsenic removal. So, during experimental work, the adsorbent properties of material were studied by determining the adsorption capacity for arsenic ions. Maximum adsorption capacity was determined by keeping in contact a well measured quantity of adsorbent material (0.1 g) with a volume of 25 mL of solutions with variable arsenic content (10, 50, 75, 100, 250, 50, 1000, 1500, 2000 μg As(V) L^−1^), for one hour in a Julabo bath shaker with 200 rotations per minute. The stock solution of arsenic ions was prepared using a standard solution containing 1000 mg As (V) L^−1^ in 2% nitric. Arsenic residual concentration in solution resulted after the adsorption was determined using mass spectroscopy inductively coupled plasma (ICP-MS Bruker Aurora M90).

Material adsorption capacity q (expressed as mg g^−1^) was calculated using the following equation:

(8) $$q=\frac{\left(C_{0}-C_{f}\right)\;V}{m}$$

where: C_o—_initial concentration of arsenic (V) from solution, (μg L^−1^); C_f—_the residual arsenic concentration (V) from solution, (μg L^−1^); V—volume of solution, (L); m—mass of adsorbent material, (g).

## 4. Conclusions

The main goal of this paper was to develop a new class of composite materials containing iron oxide grafted onto a carbon particles surface, as well as to test the obtained composite material as adsorbent for removal of arsenic from aqueous solutions.

The synthesis route involved the deployment of processes of which the temperature at which they were conducted presented a high degree of importance. In this case the composite material was obtained using as raw materials starch and ferric chloride. A high enough temperature is necessary in order to promote starch acidic hydrolysis, but it is really important to not exceed 55 °C in order to avoid gelatinization of the reaction mass. Gelatinization affects the stabilization of the formed hydro–aqua ions produced from ferric chloride hydrolysis. The stirring time represents another important parameter, because a longer reaction time can also lead to mass gelatinization which promotes migration of the iron compounds during the paste drying process, leading to separation of the used raw materials.

Based on experimental data it can be concluded that it is possible to obtain a composite material containing iron oxide particles grafted onto carbon particles. The produced iron oxide (magnetite) particles present a low size distribution, being homogenous, and having a low size.

The optimal synthesis parameters are as follows: synthesis temperature 45 °C, stirring time 240 min, thermal decomposition of synthesized paste 400 °C, heating speed rate of 5 °C min^−1^, in a nitrogen atmosphere. Such a material can be used as an efficient adsorbent for the removal of arsenic ions from water solutions. The maximum adsorption capacity has a value of 261 μg As(V) per gram, higher then the majority of the adsorption capacities found in the scientific literature.

## Figures and Tables

**Table 1:** Sample synthesis conditions.

Sample Name	Stirring Time [min]	Synthesis Temperature [°C]	Thermal Decomposition Temperature [°C]
S 1	45	55	600
S 2	240	45	600
S 3	20	58	600

**Table 2:** Maximum adsorption capacity.

Materials	Adsorbtion Capacity, q (µgAs(V)/g)	References
S1	261	Present paper
S2	160	Present paper
S3	26	Present paper
Waste rice husk	7	[46]
Rice polish	79	[47]
Human hair	12	[48]
Natural laterite	147	[49]
Clinoptilolite–rich tuff	50	[50]
Hematite	202	[51]
Montmorillonite	64	[52]
Illite	52	[52]
Activated carbons modified with iron hydro (oxide) nanoparticles	25	[53]
Nano-aluminum doped manganese copper ferrite polymer composite	50	[54]
Iron oxide coated sand	43	[55]
Granular ferric hydroxide (GFH)	4	[56]
Al_2_O_3_/Fe(OH)_3_	90	[57]
Untreated GAC (granular activated carbon)	38	[58]
